# Cholesterol Dependent Uptake and Interaction of Doxorubicin in MCF-7 Breast Cancer Cells

**DOI:** 10.3390/ijms14048358

**Published:** 2013-04-16

**Authors:** Petra Weber, Michael Wagner, Herbert Schneckenburger

**Affiliations:** Institut für Angewandte Forschung, Hochschule Aalen, Anton-Huber Str. 21, 73430 Aalen, Germany; E-Mails: petra.weber@htw-aalen.de (P.W.); michael.wagner@htw-aalen.de (M.W.)

**Keywords:** cancer cells, doxorubicin, cholesterol, fluorescence lifetime imaging microscopy (FLIM)

## Abstract

Methods of fluorescence spectroscopy and microscopy—including intensity and lifetime (FLIM) images—are used to examine uptake, intracellular location and interaction of the chemotherapeutic drug doxorubicin in MCF-7 human breast cancer cells as a function of cholesterol content. By comparing cells with natural and decreased cholesterol levels after 2 h or 24 h incubation with doxorubicin, we observed that higher fluorescence intensities and possibly shortened fluorescence lifetimes—reflecting increased uptake of the drug and more pronounced drug response—are concomitant with higher membrane fluidity.

## 1. Introduction

Doxorubicin, an anthracycline antibiotic, is used as a cytostatic drug in cancer chemotherapy, such as breast cancer, bronchial carcinoma and lymphoma, and has been studied for several decades [1,2]. The drug is taken up by cells due to passive diffusion through their membrane and finally intercalates in DNA strands, where it causes chromatin condensation and initiates apoptosis [3]. Due to its fluorescence properties [4] doxorubicin can be localized within the cells, e.g., by wide-field microscopy, and, furthermore, fluorescence lifetime measurements [5–8] permit assessing intermolecular interactions with its microenvironment. Low or moderate light doses are needed to avoid phototoxic effects in microscopic experiments [9].

In the last years various approaches for improvement of chemotherapy have been used, including encapsulation of chemotherapeutic drugs [10] or combination therapy with sensitizing substances for apoptosis [11]. If a free drug, e.g., doxorubicin, is applied, its cellular uptake may depend on membrane properties [12–15], in particular on cholesterol content which has been shown to have a high impact on membrane stiffness and fluidity [16,17]. To evaluate this impact, intracellular cholesterol was modified in the present paper with reference to a well known protocol [18]. Using this protocol, cholesterol content in U373-MG glioblastoma cells was previously reduced by about 50% upon application of 4 mM methyl-β-cyclodextrin (MβCD) [16]. “Untreated” and “cyclodextrin treated” cells will be distinguished further.

In the present manuscript fluorescence spectroscopy of suspensions of MCF-7 human breast cancer cells is combined with microscopic measurements of fluorescence images (including fluorescence lifetime images, FLIM) as well as fluorescence decay kinetics of MCF-7 cell monolayers located on an object slide. A low concentration and two different incubation times (2 and 24 h) of free doxorubicin (2 μM) are chosen in order to examine early steps of apoptosis with (almost) unchanged cell morphology.

## 2. Results and Discussion

### 2.1. Intensity of Doxorubicin Fluorescence Increases after Cholesterol Depletion

**Figure 1:**
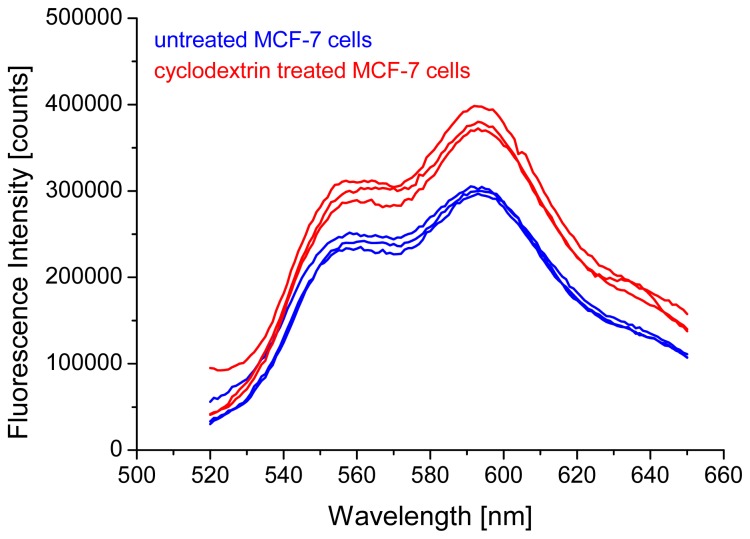
Fluorescence spectra of MCF-7 cells incubated with doxorubicin (2 μM, 24 h); spectra of three suspensions of 10^6^ cells each; untreated cells (lower curves) and cyclodextrin treated cells (upper curves) obtained during one series of experiments are compared; excitation wavelength: λ_ex_ = 470 nm.

To examine cholesterol dependent cellular uptake of doxorubicin, we determined fluorescence intensity of untreated and cyclodextrin treated MCF-7 cells, after incubation with doxorubicin (2 μM) for 24 h. Fluorescence spectra are depicted in Figure 1 for three independent measurements of cell suspensions (1 × 10^−6^ cells) in each case. Obviously, the untreated cells show lower fluorescence intensities than cells upon cholesterol depletion.

**Figure 2:**
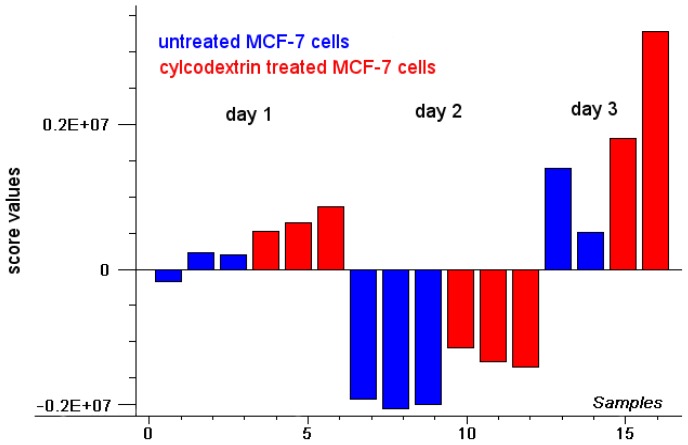
Principal component analysis (PCA); scores plot of PC1 (fluorescence intensity) for a set of 16 individual spectra obtained from three series (3 days) of experiments with samples of untreated and cyclodextrin treated MCF-7 cells.

Two further series of experiments were performed with MCF-7 cells, and although fluorescence intensity generally varied between these series, it was always lower for untreated, in comparison with cyclodextrin treated cells. For a common evaluation of all fluorescence spectra, the method of principal component analysis (PCA), a multivariate statistical method [19,20] was used. This method reduces multi-dimensional data into a few principal components which constitute a new, lower dimensional coordinate system for describing the fluorescence spectra. Common information explaining as much of the spectral variation as possible is summarized in the principal components (PCs), each one being represented by a loading spectrum and score values describing the individual spectra in the new PC coordinate system. According to PCA, 98% of spectral information was given by fluorescence intensity (PC 1) with a loading plot representing the mean fluorescence spectrum. The scores depicted in Figure 2 quantify all individual fluorescence spectra related to PC1, *i.e.*, negative scores describe spectra of lower fluorescence intensity, whereas positive scores describe spectra of higher fluorescence intensity in comparison with the mean spectrum. Figure 2 proves that fluorescence intensities were different for the three series of experiments, but in each case the scores were either more positive or less negative for the cyclodextrin treated cells in comparison with the untreated controls, thus proving higher fluorescence intensity after cholesterol depletion. It is assumed that after cholesterol depletion cell membranes were more fluid, and that the uptake of doxorubicin was, therefore, enhanced.

### 2.2. Fluorescence Lifetime Decreases as a Function of Doxorubicin Incubation Time and Cholesterol Content

**Figure 3:**
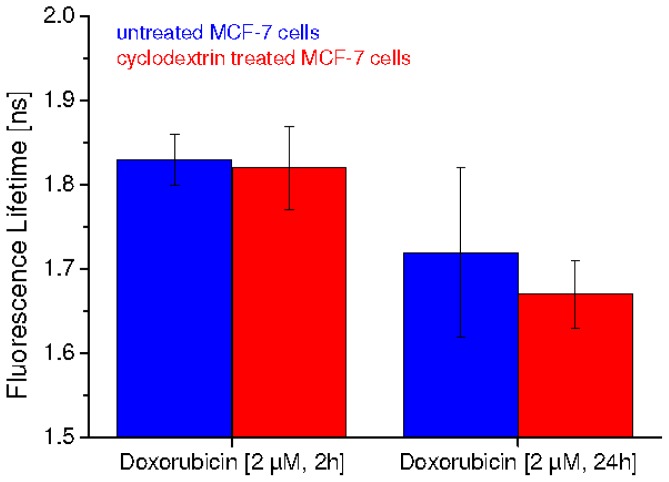
Fluorescence lifetime of untreated (blue bars) or cyclodextrin treated (red bars) MCF-7 cell monolayers after incubation with doxorubicin (2 μM) for 2 h or 24 h, respectively. Excitation wavelength: λ_ex_ = 470 nm; medians and median absolute deviations (MADs) of 15 measurements (untreated cells) or eight measurements (cyclodextrin treated cells) are indicated.

Fluorescence lifetimes of MCF-7 breast cancer cells upon 2 h or 24 h incubation with doxorubicin (2 μM) are depicted in Figure 3. While fluorescence lifetimes of untreated and cyclodextrin treated cells were almost the same (1.83 ± 0.03 ns and 1.82 ± 0.05 ns, respectively) after 2 h incubation with doxorubicin, they decreased to 1.72 ± 0.10 ns for untreated and 1.67 ± 0.04 ns for cyclodextrin treated cells after 24 h incubation. Decrease in fluorescence lifetime may result from apoptosis, as earlier reported for HeLa cells [5]. This decrease appeared to be more pronounced upon cholesterol depletion by MßCD, possibly due to an increased uptake of doxorubicin and, consequently, a more rapid apoptotic process. A non-directional Mann-Whitney *U* test with a level of significance α = 5% proved that the decrease of fluorescence lifetimes between 2 and 24 h incubation was not significant for untreated cells, but significant for cyclodextrin treated cells. Also the difference of fluorescence lifetimes at 24 h incubation between untreated and cyclodextrin treated cells revealed to be non-significant; however a tendency towards shortened fluorescence lifetimes upon cyclodextrin treatment can be deduced from Figure 3.

### 2.3. Images

**Figure 4:**
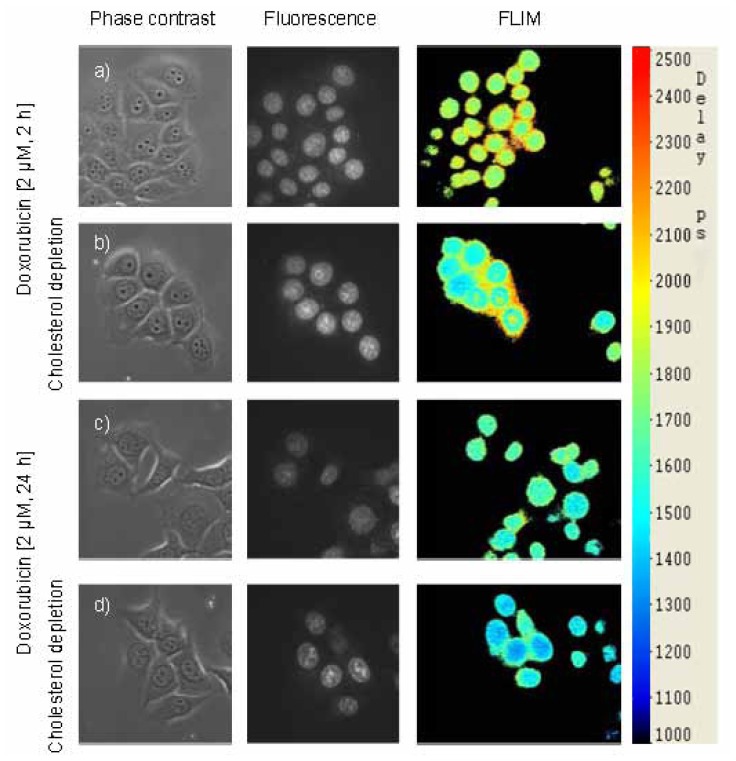
Phase contrast images, fluorescence intensities and effective fluorescence lifetimes τ_eff_ in picoseconds (from left to right) of MCF-7 cells incubated with doxorubicin (2 μM) for 2 h (**a**,**b**) or 24 h (**c**,**d**); untreated cells (**a**,**c**) and cyclodextrin treated cells (**b**,**d**) are compared. Excitation wavelength: λ_ex_ = 470 nm; fluorescence recorded at λ ≥ 520 nm; image size: 140 μm × 140 μm each.

In Figure 4 phase contrast, fluorescence intensity and fluorescence lifetime (FLIM) images of untreated and cyclodextrin treated MCF-7 cells incubated for 2 or 24 h with doxorubicin (2 μM) are depicted. While fluorescence of doxorubicin is well located in the cell nucleus, its lifetime shows a similar behaviour as depicted in Figure 3 with a decrease in fluorescence lifetime after 24 h incubation, which was more pronounced in the case of reduced (after cyclodextrin treatment) than in the case of natural cholesterol content. This indicates possible changes of intermolecular interaction, e.g., upon DNA strand breaks [5] and proves the potential of FLIM measurements for detection of these changes in processes like apoptosis.

The observed decrease of fluorescence lifetime of intracellular doxorubicin as a function of the incubation time is in agreement with the literature and indicates beginning apoptosis [5–7]. In addition, we could show that the uptake of doxorubicin is enhanced and that the process of apoptosis may be accelerated, if membrane fluidity is increased upon cholesterol depletion. This indicates that biophysical properties may have some impact on the uptake and the efficiency of chemotherapeutic drugs. For a more quantitative analysis of apoptosis, a well established sensor system, as described e.g. in [21,22] appears to be useful, and morphological studies, e.g. by scattering microscopy with angular or spectral resolution [23], may provide further information.

In a further step towards clinical application, cell monolayers may be replaced by 3-dimensional cell cultures, whose physiology, morphology and nutrient supply is closer to the *in vivo* situation in tumors [24]. Methods of 3D microscopy, e.g., laser scanning microscopy [25,26], structured illumination microscopy [27,28] or light sheet microscopy [29,30] are available for those investigations, and microfluidic systems (see e.g., [31]) may be used for application of appropriate drug doses.

## 3. Experimental Section

### 3.1. Materials

MCF-7 human breast cancer cells were obtained from Cell Lines Service (CLS No. 00273, Eppelheim, Germany). Cells were routinely grown in DMEM/HAM F-12 medium supplemented with 10% fetal calf serum and antibiotics at 37 °C and 5% CO_2_. Water soluble methyl-ß-cyclodextrine (MßCD) as well as the antitumor antibiotic doxorubicin hydrochloride was obtained from Sigma-Aldrich (München, Germany). Doxorubicin was prepared as a 2 μM stock solution in ethanol. After seeding 200 cells/mm^2^, cells were grown on microscope object slides for 48 h prior to incubation with doxorubicin (2 μM). Part of the cells was first incubated with MßCD (4 mM, 15 min) in culture medium without serum for cholesterol depletion. Subsequently cells were incubated with doxorubicin in culture medium for 2 or 24 h at 37 °C. Cholesterol depletion after application of MßCD is well documented in the literature [18]. For spectroscopic measurements cells were seeded in culture flasks, and incubated with MßCD and doxorubicin as described for the cells on object slides. After doxorubicin incubation cells were detached using trypsin/EDTA. After centrifugation and removing the supernatant, the cell pellet was re-suspended in Earl’s Balanced Salt Solution (EBSS). 1 × 10^6^ cells in a volume of 1.5 mL EBSS were transferred to a glass cuvette.

### 3.2. Experimental Setup

A diode laser with high repetition pulses (LDH 470 with driver PDL 800-B, Picoquant, Berlin, Germany; wavelength: 470 nm; pulse energy: 12 pJ, pulse duration: 55 ps, repetition rate: 40 MHz; average power: 0.5 mW) was adapted to a fluorescence microscope (Axioplan 1, Carl Zeiss Jena, Germany) by fibre optics for epi-illumination of whole cells. Fluorescence images were recorded by an electron multiplying (EM-) CCD camera with Peltier cooling and a sensitivity below 10^−16^ W/Pixel (DV887DC, ANDOR Technology, Belfast, UK) [32] using a long pass filter for λ ≥ 520 nm. For fluorescence decay kinetics and lifetime images (FLIM) a time-gated image intensifying camera (Picostar HR 12; LaVision, Göttingen, Germany) with a temporal resolution of 200 ps was used in a sampling mode (time range: 8 ns; exposure time: 1 second per channel). Data were fitted as mono-exponential curves [33], and median values as well as median absolute deviations (MADs) of fluorescence lifetimes were determined. Differences were examined by a non-directional Mann-Whitney *U* test [34] for non-normal distributions of experimental values with a level of significance α = 5%. A grating spectrometer (Jobin Yvon, JY.3 447) operated at a spectral resolution of 10 nm was used for recording fluorescence spectra of cell suspensions in a glass cuvette. A commercial program (Unscrambler 9.8; Camo process As, Oslo, Norway) was used for Principal Component Analysis.

## 4. Conclusions

As demonstrated above, a combination of fluorescence spectroscopy, fluorescence imaging and fluorescence decay kinetics may be useful to measure cellular uptake, intracellular distribution and intermolecular interactions of the chemotherapeutic drug doxorubicin in cancer cells prior and during apoptosis. In particular, uptake of doxorubicin and drug response (assessed by changes in fluorescence lifetime) were examined in the context of cholesterol dependent membrane fluidity. Cholesterol content is suggested to be considered for future application of chemotherapeutic drugs.
